# Epigenetic regulation of sex dimorphism in cardiovascular health

**DOI:** 10.1139/cjpp-2023-0406

**Published:** 2024-03-01

**Authors:** Charan Thej, Raj Kishore

**Affiliations:** a Aging and Cardiovascular Discovery Center, Lewis Katz School of Medicine, Temple University, Philadelphia, PA, 19140, USA;; b Department of Cardiovascular Sciences, Lewis Katz School of Medicine, Temple University, Philadelphia, PA 19140, USA

**Keywords:** epigenetics, cardiovascular, sex dimorphism

## Abstract

Cardiovascular diseases (CVDs) remain the leading cause of morbidity and mortality, affecting people of all races, ages, and sexes. Substantial sex dimorphism exists in the prevalence, manifestation, and outcomes of CVDs. Understanding the role of sex hormones as well as sex-hormone-independent epigenetic mechanisms could play a crucial role in developing effective and sex-specific cardiovascular therapeutics. Existing research highlights significant disparities in sex hormones, epigenetic regulators, and gene expression related to cardiac health, emphasizing the need for a nuanced understanding of these variations between men and women. Despite these differences, current treatment approaches for CVDs often lack sex-specific considerations. A pivotal shift toward personalized medicine, informed by comprehensive insights into sex-specific DNA methylation, histone modifications, and non-coding RNA dynamics, holds the potential to revolutionize CVD management. By understanding sex-specific epigenetic complexities, independent of sex hormone influence, future cardiovascular research can be tailored to achieve effective diagnostic and therapeutic interventions for both men and women. This review summarizes the current knowledge and gaps in epigenetic mechanisms and sex dimorphism implicated in CVDs.

## 1. Introduction

Cardiovascular diseases (CVDs) remain a predominant cause of illness and death globally, affecting individuals across diverse demographics. Notably, sex disparities play a significant role in the prevalence, manifestation, and outcomes of these diseases, revealing a complex interplay of factors that extend beyond sex hormones. Sex hormones influence sex dimorphism in cardiovascular health by modulating vascular function (Akishita and Yu 2012), inflammation (Corcoran et al. 2010), oxidative stress (Xiang et al. 2021), and lipid metabolism (Palmisano et al. 2018). However, the risk of CVD-related deaths is one-third in women as compared to men at all ages, indicating that sex hormone-independent mechanisms may also contribute to higher survival rates in women (Benjamin et al. 2017; Roth et al. 2018; Virani et al. 2020; Regitz-Zagrosek and Gebhard 2023). Several epigenetic modifications, including DNA methylation, histone modifications, and non-coding RNA, regulate the profound effects of gene expression, cellular functions, and disease (Feinberg 2018; Wu et al. 2023). This review aims to explore the current understanding and gaps in our knowledge concerning these epigenetic mechanisms and the sex dimorphism in CVDs. This review provides a nuanced and personalized approach to cardiovascular research and therapeutics that transcends traditional sex boundaries.

## 2. Epigenetic mechanisms in cardiovascular health and disease

### 2.1. DNA methylation

DNA methylation involves the transfer of a methyl group to the C5 position of cytosine to form 5-methylcytosine (5mC), which further recruits proteins involved in gene repression or inhibiting transcription factor binding (Mattei et al. 2022). CpG dinucleotides facilitate docking sites for methyl groups to determine variation in DNA methylation (Moore et al. 2013). In 1925, 5mC was first discovered in bacteria (Johnson and Coghill 1925); however, its function was unknown. The term “epigenetics” was coined by C. Waddington in 1940 to describe heritable changes in genes independent of changes to the DNA sequence, and DNA methylation was found to be the key mechanism. Later, Rollin Hotchkiss found modified cytosine in paper chromatography of calf thymus DNA and predicted it to be 5mC (Hotchkiss 1948). Holliday and Pugh proposed that two enzymes orchestrate de novo maintenance and methylation-regulated switching genes on and off (Holliday and Pugh 1975), which was correlated to the transfer of somatic nuclear transfer to an enucleated oocyte by John Gurdon (Gurdon and Uehlinger 1966). Most importantly, sex dimorphism due to X-chromosome inactivation in males is orchestrated by DNA methylation. Differential DNA methylation extends to various tissues and organs causing phenotypic and metabolic differences between the sexs resulting in differential disease pathogenesis and progression (Rinn and Snyder 2005).

DNA methylation involves enzymatic catalysts broadly classified as writers, erasers, and readers. Primarily, DNA methylation writers are DNA methyltransferases (DNMTs). Among them, DNMT1 is a maintenance methyltransferase responsible for replicating the existing methylation pattern during DNA replication, ensuring the inheritance of the methylation pattern from the parent strand. En Li et al. showed that DNMT1 knockout (KO) in embryonic stem cells reduced overall DNA methylation but did not influence their proliferation (Li et al. 1992). However, DNMT1-KO embryos showed abnormal development and embryonic lethality (Li et al. 1992), confirming the role of DNMT1 as a maintenance methyltransferase. In the context of CVDs, DNA hypomethylation has been linked to atherosclerosis (Hiltunen et al. 2002; Zhang et al. 2021) and inflammation (Makar and Wilson 2004; Yi 2021). The role of DNMT1 in heart disease remains ambiguous. Depletion of DNMT1 in a rat model of heart failure significantly improved cardiac functions (Wu et al. 2020). In contrast, another study showed that high expression of DNMT1 suppresses miR152–3p, causing downregulation of ETS1 and RhoH, decreasing mitophagy and autophagy, and aggravating heart failure (Deng et al. 2022). However, the role of DNMT1 in regulating sex dimorphisms in CVD has been insufficiently studied. Broadly, one study showed substantial differential DNA methylation patterns between male and female hearts after perinatal lead exposure (Svoboda et al. 2021). Unlike the maintenance methyltransferase DNMT1, de novo methyltransferases, DNMT3a and DNMT3b, are known to establish new patterns of DNA methylation and take part in proliferation and differentiation of embryonic stem cells, as well as in embryonic development (Okano et al. 1999; Dan and Chen 2022). The distinct roles of DNMT3a and DNMT3b have been highlighted in regulating de novo DNA methylation states in hematopoietic and epidermal stem cells to result in varied fates (Challen et al. 2014; Rinaldi et al. 2016). A study showed that DNMT3a and 3b are dispensable for regulating cardiac function in a double knockout pressure overload mice model (Nührenberg et al. 2015). In contrast, another study found that knockout of DNMT3a in human cardiomyocytes (CMs) resulted in higher atrial gene expression and lower MYH7/MYH6 along with dysregulation of HIF-1*α* and aberrant activation of glucose and lipid metabolism, causing lipid vacuole accumulation (Madsen et al. 2020). Rauch PJ et al. showed that the loss of DNMT3a in myeloid cells enhanced inflammation and increased atherosclerosis in mice, highlighting the importance of de novo methyltransferases in cardiac physiology and pathogenesis (Rauch et al. 2023). Some studies identified that DNMT3b and DNMT3L in male and female germ cells impact differential features of gamete-derived chromatin and transcription in embryos (Shirane and Lorincz 2022). Differential expression of DNMT1, DNMT3a, and DNMT3b were found in CD4 + T cells in healthy males and females, as well as in patients of systemic lupus erythematosus (Balada et al. 2008). Erasing of DNA methylation occurs using chemical reactions that modify 5mC by deamination, oxidation, or alkylation, which are recognized by the base excision repair (BER) mechanisms that replace the base with cytosine (Hegde et al. 2008; Drohat and Coey 2016). The role of DNMTs has not been sufficiently investigated in the context of sex dimorphism in cardiovascular homeostasis and disease.

### 2.2. Histone modifications

Histone modifications can be associated with sex dimorphism in cardiovascular health and disease, with or without the influence of sex hormones. Various chemical modifications on histone tails include acetylation, methylation, phosphorylation, ubiquitination, or SUMOylation. However, histone acetylation, deacetylation, methylation, and demethylation have been well-studied in the context of CVD outcomes.

Histone acetylation involves neutralizing the positive charge of histone by adding acetyl groups, weakening the interaction with negatively charged DNA, and resulting in an open chromatin structure. This process makes accessing the DNA easier for cell machinery (Barski et al. 2007). Acetylation of histones near gene promoter regions is associated with increased transcriptional activity, allowing transcription factors and RNA polymerase to bind to DNA and initiate gene expression effectively (Martin et al. 2021). Dysregulation of histone acetylation has been associated with several CVDs, contributing to their development and progression. For instance, a common histone acetyltransferase (HAT), p300/CBP, is involved in acetylating histones at GATA-4, the overexpression of which is linked to left ventricular dilation and systolic dysfunction (Yanazume et al. 2003). Moreover, p300/CBP knockout mice show defects in cardiac structural proteins such as *β* myosin heavy chain (*β*MHC) and *α*-actinin, with reduced trabeculation and die before 11.5 of gestation (Yao et al. 1998). Inhibition of p300 using a specific inhibitor C646 reversed cardiac fibrosis, hypertrophy, and cardiac function by improving coronary flow reserve in Sirtuin 3 (SIRT3) knockout mice (Su et al. 2020). Curcumin, a natural inhibitor of the HAT, p300, has been shown to reduce cardiac hypertrophy in a heart failure model (Morimoto et al. 2010). In addition to CVDs, patients with high abdominal aortic aneurism (AAA) were found to have high aortic expression levels of lysine-specific acetyltransferases (KAT) family of proteins such as KAT2A, KAT2B, KAT3A, KAT3B (p300/CBP), and KAT5–8, causing acetylation of H3K9, H3K18, and H3K14, effecting AAA diameter and blood urea nitrogen (Han et al. 2016).

Histone deacetylation is a process where the removal of acetyl groups by histone deacetylases (HDACs) results in chromatin condensation, thereby inhibiting gene transcription (Seto and Yoshida 2014). HDACs have been extensively implicated in controlling cardiovascular homeostasis and pathophysiology (Bagchi and Weeks 2019). HDACs are divided as class I (HDAC1, HDAC2, HDAC3, and HDAC8), class II (HDAC4, HDAC5, HDAC6, HDAC7, HDAC9, and HDAC10), and class III (SIRT1–7) proteins based on zinc- or NAD+ biochemical pathways to deacetylate lysine substrates (Seto and Yoshida 2014).

The overexpression of the unusual homeobox gene *Hod* is known to cause cardiac hypertrophy (Chen et al. 2002). Knocking out the expression of HDAC2 in a Hod-Tg-Hdac^−/−^ mice prevented cardiac hypertrophy by inhibiting the upregulation of hypertrophic genes, atrial natriuretic factor (ANF), Myh7 (*β*-MHC), and Acta1 (*α*-smooth muscle actin) (Trivedi et al. 2007). Inhibition of HDAC using an inhibitor, Givinostat/ITF2357, increased left ventricular by inhibiting ECM remodeling and reduced fibrosis in a model of diastolic dysfunction with preserved ejection fraction in rats (Travers et al. 2021). We have recently shown that HDAC1-mediated deacetylation of H3K9 leads to the downregulation of angiogenic, survival, and proliferative genes in cardiac endothelial cells. Importantly, we found that HDAC1 can be carried in exosomes of cells to elicit functions in target cells. Inhibition of HDAC1 in cardiac endothelial cells and their exosomes promoted robust angiogenesis in vitro, as well as in a mouse model of myocardial infarction (MI) (Huang et al. 2022).

Sex dimorphism has been observed in the regulation of HDACs influencing cardiac outcomes. One such study identified variation in heart weights between female mice and male mice fed with trans fatty acid diet, with female mice exhibiting lower cardiac weight and downregulated expression of HDAC2, leading to downregulated GATA4, MEF2D, and SREBF2 (Collison et al. 2011). However, no significant variations have been found in the roles of HDACs in regulating cardiac functions in neonates. For instance, in both males and females, HDAC1 and HDAC2 were found to be involved in regulating calcium influx, calcium handling, and contractility, and deletion of both these genes caused embryonic lethality (Montgomery et al. 2007).

Histone methylation regulates epigenetic modifications that play key roles in various cellular processes, including heterochromatin formation, gene imprinting, X chromosome inactivation, and transcriptional gene regulation (Hyun et al. 2017; Gagnidze and Pfaff 2021). Histone methylation targets specific amino acids within histones, with lysine (Lys), arginine (Arg), and histidine (His) acting as methyl acceptors. In histone H3, Lys 4, 9, 26, 27, 36, 56, and 79, as well as Arg 2, 8, and 17, undergo methylation, resulting in altered chromatin structure. Histone H4 has fewer methylation sites, including Lys 5, 12, and 20 (Hyun et al. 2017). However, modifications in histone H2A and H2B methylation within the histone octamer are not well studied. For instance, the SYMD family of histone methyltransferases (SMYD1–5) share highly conserved structural and functional domains SET and MYND (Tracy et al. 2018). Interestingly, SMYD1 is primarily expressed in skeletal and cardiac muscles, rendering it crucial for myogenic differentiation and myosin assembly. Deletion of SMYD1 disrupts cardiac differentiation and maturation, leading to embryonic lethality (Du et al. 2014). A recent study found that carnitine palmitoyltransferase CPT1B knockout mice had a 20-fold accumulation of *α* ketoglutarate (*α*KG) and highlighted the role of *α*KG in activating KDM5 leading to the demethylation of H3K4me3 at genes essential for maintaining CM proliferation and growth (Li et al. 2023). The histone H3K27me3 methyltransferase Ezh2-mediated repression of sine oculus-related homeobox 1 (SIX1) in cardiac progenitors is necessary for maintaining homeostasis in postnatal hearts (Delgado-Olguín et al. 2012). The loss of Dot1l, which mediates H3K79 methylation, results in dilated cardiomyopathy with chamber dilation and systolic dysfunction. Moreover, Dotl1 was significantly lower in human idiopathic dilated cardiomyopathy (Nguyen et al. 2011). Another study found that reduction of H3K9me3 by inhibiting the heterochromatin protein 1 (HP1) using the methyltransferase inhibitor, chaetocin significantly decreased progression of heart failure by regulating mitochondrial PGC1*α* in a rat model of high salt-induced heart failure (Ono et al. 2017).

Interestingly, histone modifications have also been shown to be regulated by non-coding RNAs such as lnRNA and miRNA.

### 2.3. Non-coding RNAs

Non-coding RNAs essentially do not code for a particular protein; however, this does not mean they are not functional. Non-coding RNAs (ncRNA) include microRNA (miRNA), long non-coding RNA (lnRNA), small-nucleolar RNA (snoRNA), PIWI-interacting RNA (piRNA), large-intergenic non-coding RNA (lincRNA), circular RNA (circRNA), and transcribed ultra-conserved regions (T-UCR), which may contribute to homeostasis and disease progression (Esteller 2011; Nemeth et al. 2024).

NcRNAs have been shown to play key roles in cardiovascular health and disease. Strikingly, MiR-1 is exclusively expressed in skeletal and cardiac muscle regulating development, contractility, and disease (Zhao et al. 2005). It was shown that miR-1 genes are direct transcriptional targets of muscle differentiation regulators, particularly serum response factors MyoD and Mef2. Moreover, overexpression of MiR-1 in developing hearts resulted in decreased pool of ventricular CMs (Zhao et al. 2005). A total of nine miRNAs are putatively regulated by MyoD/myogenin and have been shown to be muscle-specific (Rao et al. 2006). Similarly, several lncRNA have been implicated in several CVDs (Hobuß et al. 2019). Han et al. found a cluster of lncRNA transcripts from the myosin heavy chain 7 (Myh7) loci, called myosin heavy chain-associated RNA transcript (Mhrt), and demonstrated the lncRNA–chromatin-linked mechanism of cardiac hypertrophy. They further demonstrated that hypertrophy was due to repression of Mhrt by Brg-1-hdac-parp repressor complex activated due to pathological stress (Han et al. 2014). Studies have shown differential regulation of snoRNAs in hypertrophic cardiomyopathy (HCM), specifically in HCM-derived extracellular vesicles. In a model of induced pluripotent stem cell (iPSC)-derived HCM extracellular vesicles, James V et al. found that 10 snoRNAs were enriched in the HCM iPSC CM-EVs, including SNORD166, multiple paralogs of which were detected (James et al. 2021). SNORD116 is typically expressed in brain tissue, but is also found in other tissues such as heart, ovary, prostate, thyroid, and kidney (Galiveti et al. 2014). Likewise, piRNAs are abundantly expressed in the heart; however, their functions during the event of CVDs are poorly explored. A piRNA called CHAPIR was found to be regulating cardiac hypertrophy by targeting METTL3, an RNA methyltransferase responsible for N^6^-methyladenosine (m6A) methylation of mRNA transcripts, leading to upregulation of PARP10 (Gao et al. 2020). Another piRNA has been implicated in promoting CM apoptosis by regulating RNA N^4^ acetyl cytidine (ac^4^C) modifications. Specifically, another piRNA HAAPIR enhances acetylation modifications on TFEC mRNA by recruiting NAT10, an RNA acetyltransferase. The acetylation stabilizes TFEC mRNA, increases its translational efficiency, and subsequently elevates the expression of TFEC, which in turn promotes transcription of BIK, a pro-apoptotic factor leading to CM death (Wang et al. 2022). A previously uncharacterized lincRNA named Lionheart was found to be abundantly expressed in the striated muscle, especially in the heart and is upregulated during pathological cardiac remodeling (Kuwabara et al. 2020). Knockout of Lionheart in mice reduced Myh6, which was linked to the interaction between Lionheart and purine-rich element binding protein A (PURA), preventing PURA from binding to purine-rich negative regulatory (PNR) element at the Myh6 locus (Kuwabara et al. 2020).

CircRNAs have been reported as significant regulators in CVDs, particularly in MI. Over 1200 circRNAs have been consistently detected in human, mouse, and rat tissues with several functional implications (Werfel et al. 2016). In the context of CVDs, a heart-related circRNA (HRCR) was found to be acting like a sponge for endogenous miR-223 to inhibit cardiac hypertrophy and heart failure (Wang et al. 2016). Our lab found a circRNA named circFndc3b to be significantly repressed in post-MI in mice and human ischemic cardiomyopathy patients (Garikipati et al. 2019). Overexpression of CircFndc3b was found to inhibit cardiac apoptosis and induce angiogenesis in a FUS-VEGF-dependent manner (Garikipati et al. 2019).

Most of these studies did not evaluate the aspect of sex dimorphism. Some evidence exists that X-chromosome-linked miRNAs may well contribute to the molecular mechanisms of cardiovascular protection in women (Florijn et al. 2018). One study showed that sex-specific expression of miR-222 was found in myocardium, which was proposed to regulate sex-dimorphic cardiac eNOS expression (Evangelista et al. 2013). More studies are necessary to demonstrate the sex-specific roles of non-coding RNAs.

The role of various epigenetic modifiers and their sex-specific roles in CVD are summarized in Table 1.

**Fig. 1.**
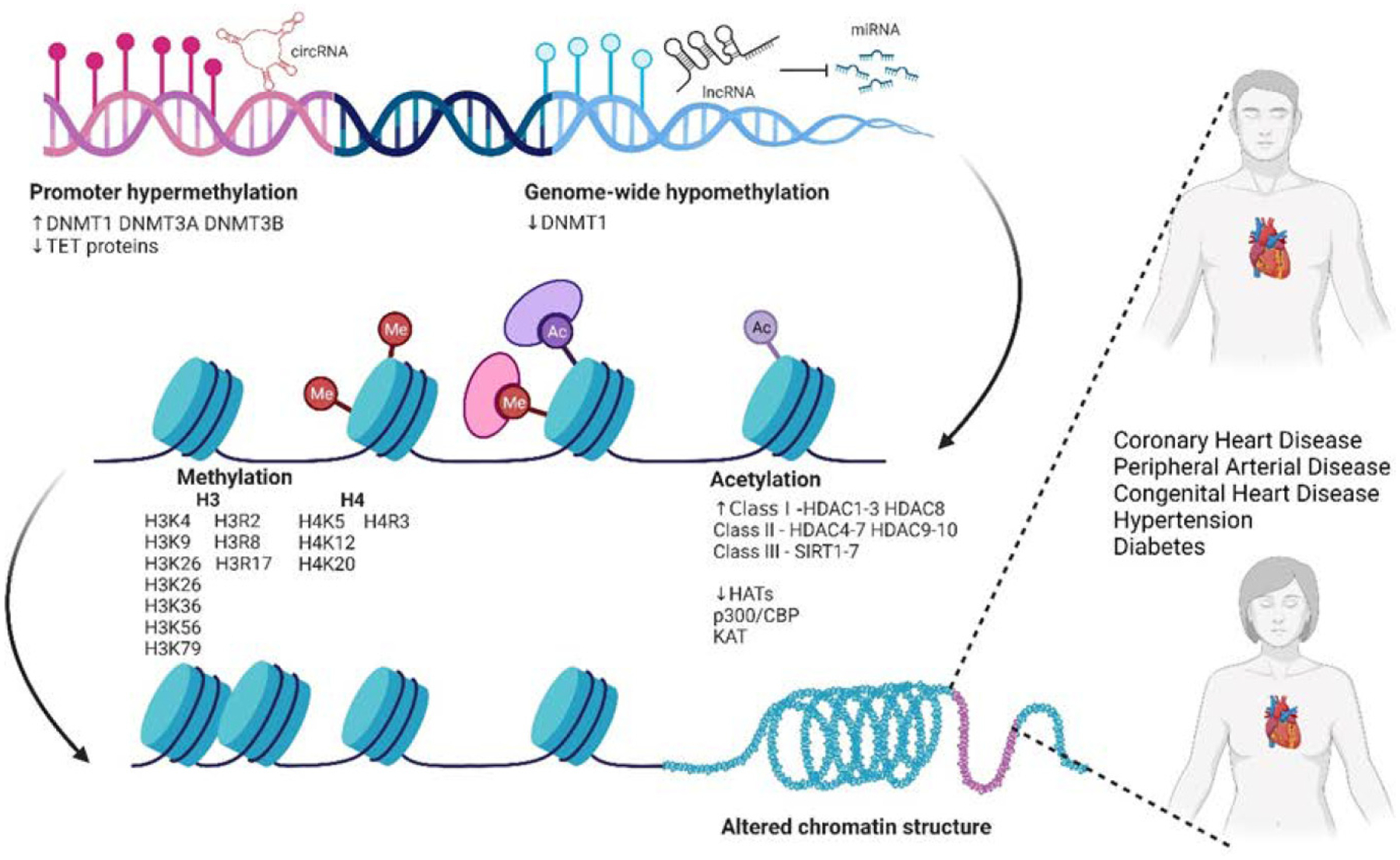
Epigenetic regulation of sex dimorphism in cardiovascular disease. DNA methylation, non-coding RNAs, and histone modifications contribute to altered chromatin structure, resulting in diverse presentations of cardiovascular diseases in both men and women. Figure was created using BioRender.

The epigenetic mechanisms of sex dimorphism are succinctly summarized in Fig. 1, highlighting the responses of DNA methylation, histone modifications, and the roles of non-coding RNA.

## 3. Sex dimorphism in cardiovascular pathologies

Most studies have focused on men, and there is a need for comprehensive research into the sex-specific risk factors associated with cardiovascular pathologies.

Atherosclerosis is the leading cause of most CVDs and often presents differently in men and women. Atherosclerosis manifests due to an imbalance in lipid metabolism, oxidation of low-density lipoproteins gradually progressing to inflammation (Libby et al. 2019; Salekeen et al. 2022). Either oxidized LDL or LDL-C can get deposited within the intima of the arterial walls, provoking a series of adverse cellular and molecular functions such as the synthesis of ECM molecules such as collagens, elastins, and other proteoglycans, triggering an inflammatory response by macrophages and other immune cells (Libby et al. 2019; Jiang et al. 2022). In general, the incidence of atherosclerosis occurs earlier in men compared to women. However, the incidence and deaths related to atherosclerosis dramatically increase in postmenopausal women, suggesting the protective role of sex hormones (Man et al. 2020). Carotid artery intima-media thickness (IMT), a reliable parameter for early atherogenesis, was higher in boys than girls (Böhm et al. 2009; Zanini et al. 2019). Similar observations were made in middle-aged people (Diaz et al. 2018). A study involving 5341 individuals who underwent carotid artery ultrasonography found varied echogenicity of plaques between men and women. They found that all inflammatory marks were associated with plaque size in men. In contrast, white blood cell (WBC) was associated with plaque echogenicity in women, highlighting sex-dependent differences in associations of carotid atherosclerosis and inflammatory markers (Halvorsen et al. 2009). Several studies have highlighted the importance of epigenetics, particularly DNA methylation, in the progression of atherosclerosis. DNA methylation is regulated by DNMTs (DNMT1, DNMT3a, and DNMT3b) and reversed by TET methyl cytosine dioxygenases (TET1, 2, and 3) (Gu et al. 2018). A study found that TET2 is essential for inhibiting NLRP3-inflammasome-mediated IL-1*β* secretion associated with clonal hematopoiesis and highlighting its athero-protective role (Fuster et al. 2017). Another study supported these findings in a cohort of 4726 participants with coronary heart disease and 3529 controls (Jaiswal et al. 2017). Whole-exome sequencing showed a significant association between clonal hematopoiesis of indeterminate potential (CHIP) and coronary heart disease, particularly associated with TET2 mutation. Moreover, transplantation of bone marrow (BM) from TET2^−/−^ mice into atherosclerosis-prone Ldlr^−/−^ mice and feeding them high cholesterol diets induced significantly larger lesion size compared to the control group (Jaiswal et al. 2017). Sex-specific associations between DNA methylation and atherosclerosis are insufficiently investigated. Conversely, a study showed that hypermethylation of the ABCG1 gene at the promoter region was significantly associated with ischemic stroke, and this relationship was notably stronger in women. Hypermethylation of ABCG1 gene was also linked to atherosclerosis with increased carotid-intima media thickness, especially in women (Qin et al. 2019), highlighting the importance of epigenetics in disease-associated sex dimorphisms.

Hypertension is a widespread public health crisis with substantial racial, ethnic, and sex disparities in its prevalence, treatment, and control rates worldwide. Hypertension is a preventable risk factor for CVDs on a global scale. The prevalence of hypertension, characterized by systolic and diastolic blood pressure (BP) values above specific thresholds (American Heart Association—130/80; European Society of Cardiology—140/90), has been steadily increasing due to factors such as aging, unhealthy lifestyles, and insufficient physical activity (Staessen et al. 2003; Mills et al. 2020). A recent analysis of BP trends in a large cohort of 32 833 individuals showed a significant sex-dependent pattern in BP trajectories, with lower hypertension in women compared to men over 60, where the patterns converge (Ji et al. 2020). Interestingly, females in this group experienced a sharper incline after age30. However, it was unclear whether these differences were due to sex hormones, genetics, epigenetics, or sex (Ji et al. 2020). One crucial factor contributing to accelerated BP trajectory in females may be the impact of hypertensive disorders during pregnancy. A study of 58 671 females from the Nurses’ Health Study II, with no history of who has no history of hypertension or CVDs, revealed that gestational hypertension and pre-eclampsia during first pregnancy were linked to a two-fold increase in chronic hypertension (Stuart et al. 2018).

Adverse Cardiac Remodeling in MI encompasses hemodynamic challenges and unfavorable prognostic implications such as a proinflammatory response, increased wall stress, and neurochromal activation sequestered by underlying epigenetic regulators (Zhao et al. 2022). The impact of MI is compounded by changes in the infarct border zone, remote myocardium, and systemic responses that culminate into heart failure. Moreover, neurohormonal activation, including angiotensin II, aldosterone, and norepinephrine, promotes cardiac and renal dysfunction (Hartupee and Mann 2017). Sex-based differences in cardiac remodeling exist, with men experiencing dilation and women developing hypertrophy (Kessler et al. 2019). Following MI, women tend to develop heart failure with preserved ejection fraction (HFpEF), characterized by impaired diastolic function and concentric hypertrophy. In contrast, men are likely to present with heart failure with reduced ejection fraction (HFrEF) associated with systolic dysfunction and hypertrophy (Tadic et al. 2019). Epigenetics plays a crucial role in pathological remodeling after ischemic cardiac injury. As discussed in the previous sections of this review, DNMTs, HDACs, HATs, and non-coding RNA have extensively been implicated in adverse remodeling after myocardial injury. However, sex-specific epigenetic dimorphisms have been insufficiently studied in the context of atherosclerosis, hypertension, or other CVDs.

## 4. Sex hormone influences and epigenetics

Sex differences in cardiac structure and function can exist in healthy hearts and pathological conditions. Research in both humans and animals revealed baseline disparities in cellular and molecular processes, such as rhythmicity, lipid metabolism, regenerative capacity, inflammation, and fibrosis between male and female cardiac cells, caused by variable levels of sex hormones (Deegan et al. 2021). The variation in CVD outcomes between males and females, although not solely associated with sex hormones, androgens, estrogens, and their receptors, plays a crucial role in regulating homeostasis or pathological responses in various cardiac cell types. Androgens, including testosterone and dihydrotestosterone (DHT), exert their effects by binding to androgen receptors (ARs) and modulating gene expression. Androgens directly interact with DNMTs influencing the methylation status of specific genomic regions. They modulate DNA methylation patterns, particularly in genes that may be associated with cardiovascular function and disease. Testosterone and its more potent derivative, DHT, engage with cytoplasmic AR under the guidance of heat shock proteins (Goodale et al. 2017). Upon binding, the complex formed by DHT and AR translocates to the nucleus, where it forms dimers with another DHT–AR complex. This dimeric structure then recruits coactivator proteins, initiating the transcriptional activation of a specific group of genes containing androgen response elements. These genetic alterations induced by the DHT–AR complex lead to modifications in the behavior of myocardial and vascular cells, influencing various cardiovascular processes (Goodale et al. 2017). Another previous study showed that ARs on CMs respond to testosterone and DHT, causing hypertrophy. Low testosterone levels in middle-aged/older men have been linked to increased CVD risk. This cross-sectional study examined 58 healthy, nonsmoking men across different age groups and testosterone levels. Results revealed that middle-aged/older men with low testosterone exhibited heightened age-associated endothelial dysfunction, partly attributed to increased oxidative stress and inflammation (Babcock et al. 2022). Inhibition of ARs using cyproterone abolished the hypertrophic response (Marsh et al. 1998). Moreover, androgen blockage has been associated with positive outcomes in CM death, disease, and stroke (Hu et al. 2020). The role of testosterone or other androgens in regulating epigenetics has been insufficiently studied in the context of CVDs.

Despite accumulating evidence supporting the protective functions of progesterone in the cardiovascular system, research in this area has been limited. Progesterone has been shown to lower BP, inhibit coronary hyperactivity, and exhibit potent vasodilatory and natriuretic effects (Thomas and Pang 2013). However, potential beneficial actions of progesterone on cardiovascular functions have been overshadowed by divergent effects observed in clinical trials with synthetic progestins, like medroxyprogesterone acetate, associated with increased coronary disease risk. Progesterone’s swift activation of second messenger pathways in vascular cells, involving both nuclear progesterone receptors (PRs) and novel membrane progesterone receptors (mPRs), suggests a crucial role in regulating vascular tone. New data reveal the presence of mPR*α*, mPR*β*, and mPR*γ* in human endothelial and smooth muscle vascular cells, suggesting their involvement in rapid progestin signaling. Preliminary evidence indicates that mPRs may mediate progesterone’s effects on cAMP production and nitric oxide synthesis, emphasizing the need for further investigation into the role of mPRs in progesterone’s regulation of cardiovascular functions (Thomas and Pang 2013). Despite progesterone level variations during the reproductive cycle, its comparable levels in males and females suggest a broader influence on cardiac physiology. Key findings from a study imply that fluctuating progesterone levels, coupled with sex-specific DNA binding of PRs, may contribute to sex-based differences in cardiovascular health (Sim et al. 2021). This research opens avenues for exploring therapeutic targeting of progesterone signaling to modify sexually dimorphic CVD outcomes. Additionally, the study highlights the profound maturation of all cardiac cell lineages during human heart development, with progesterone potentially playing a role in postnatal maturation of other cardiac lineages (Sim et al. 2021).

Similarly, when bound to estrogen, the two well-characterized estrogen receptors (ERs), ER*α* and Er*β*, get translocated to the nucleus and regulate various cellular responses in cardiac cells, such as survival, hypertrophy, inflammation, mitochondrial electron transport, etc. Conversely, ER regulates the PI3K-AKT signaling from the plasma membrane (Murphy 2011). We have previously shown that ERs on bone marrow endothelial progenitor cells (BM-EPCs) are essential for regulating their mobilization and repair during cardiac ischemic injury (Hamada et al. 2006). Both ER*α* and Er*β* knockout EPCs that incorporated into the ischemic border zone were significantly reduced compared to wild-type EPCs, with significantly downregulated expression of vascular endothelial growth factor (VEGF) leading to reduced capillary density in the ischemic myocardium (Hamada et al. 2006). However, transcriptional sex biases can exist independently of sex hormone influence. The Genotype-Tissue Expression (GTEx) project found that only a few differentially expressed autosomal genes across various human somatic tissues express androgen or estrogen response elements, indicating indirect hormonal influences or regulation by other mechanisms (Lonsdale et al. 2013). The loss of sex hormones, particularly estrogen and progesterone, in women post-menopause has been attributed to the substantially increased incidence of CVDs. Particularly, estrogen has been implicated in preventing CVD onset in women. However, hormone replacement clinical trials showed that neither estrogen nor estrogen with medroxyprogesterone acetate could significantly halt the progression of CVD (Hodis and Mack 2022). Sex-chromosome-linked gene regulation associated with cardiac function and pathology is gaining recognition. Both X and Y chromosome-linked genes can affect gene expression, particularly by epigenetic regulation. For instance, an X-chromosome-linked gene, Kdm6A/UTX, with two copies in females and one copy in males, has been implicated in protecting against CM apoptosis by demethylating H3K27me3 at the Ncx (sodium–calcium exchangers) gene. Similarly, the gene trap mouse model for the Y-chromosome-specific UTY/Ddx3y resulted in substantial genetic variation in cardiac left ventricle compared to wild-type mice, emphasizing the role of sex chromosomes in cardiac homeostasis and disease.

## 5. Sex dimorphism in stem cells

The ability of injured myocardium to recruit extra-cardiac stem cells like BM-derived stem/progenitor cells (hereto referred to as EPC) is critical to aid in ischemic repair and regeneration. Preclinical data from experimental ischemic injuries, including those from our labs, suggest that EPC participates in the process of neo-vascularization and tissue repair, leading to enhanced recovery of ischemic myocardium (Krishnamurthy et al. 2011; Thal et al. 2012; Cheng et al. 2015; Joladarashi et al. 2015; Garikipati et al. 2017). Furthermore, clinical trials involving EPC transplantation for ischemic myocardium and critical limb ischemia (CLI) have confirmed this possibility. However, the clinical outcome of such therapies was modest (Assmus et al. 2002; Strauer et al. 2002; Britten et al. 2003; Losordo et al. 2007). Whether sex influences EPC/stem cell reparative properties has not been well studied. Notably, there appears to be a significant sex imbalance among cardiac patients undergoing clinical BM-cell-based therapy. For example, data from clinical trials using BM progenitors for AMI patients suggest a preponderance of male patients (>80%) compared to 20% of female patients (Gyöngyösi et al. 2015; Vrtovec et al. 2018). Even preclinical animal studies reporting direct comparison of sex-specific stem/progenitor cells for their post-MI myocardial reparative properties are scant. Recently, however, evidence has begun to emerge that sex does influence the functionality of stem/progenitor cells. Mesenchymal stem cells (MSC) have been reported to exhibit sex-related differences in paracrine function, including higher production of VEGF and less secretion of TNF-*α* by female MSCs in response to stress stimuli such as lipopolysaccharide and hypoxia (Crisostomo et al. 2006), suggesting that source sex may be an essential determinant of MSC function and potential cardioprotection. Moreover, intra-coronary infusion of female MSCs was associated with greater post-ischemic myocardial functional recovery compared to male MSCs (Crisostomo et al. 2007). Male MSCs showed significantly greater TNF and IL-6 and significantly less VEGF secretion than female MSCs and female MSCs were more potent in suppression of T cell proliferation (Siegel et al. 2013). Female BM-derived MSC showed greater therapeutic efficacy than male MSC in reducing neonatal hyperoxia-induced lung inflammation and vascular remodeling (Sammour et al. 2016). Similarly, circulating EPCs display a higher colony forming and migratory activities in middle-aged women compared with age-matched men (Hoetzer et al. 2007). Transcriptome analysis on muscle biopsies have shown that female cells are enriched with genes associated with oxidative metabolism and protein catabolic pathways: processes that influence cell longevity and their ability to divide (Sammour et al. 2016). Although these studies are suggestive, a direct comparison of sex-specific EPC/other stem cells on their differential myocardial reparative properties and the mechanistic basis of such functional dimorphisms, particularly estrogen-independent mechanisms, is lacking.

Numerous studies have established a cardio-protective role of estrogens in models of exogenous estrogen supplementation following surgically induced menopause/ovariectomy (OVX). Similarly, the role of estrogen/ER on female EPC function has also been reported extensively. 17-beta estradiol (E2), the most active estrogen produced by ovaries, enhanced mobilization, survival, and promotion of re-endothelialization of female EPCs in mice with vessel injury (Herrmann et al. 2011). A clinical study also showed higher proangiogenic potential of fertile females compared to age-matched males (Stampfer et al. 1991). Several previous studies from our labs have also demonstrated that E2 supplementation enhanced EPC-mediated ischemic heart repair mainly via ER*α* and in OVX mice via mechanisms involving eNOS upregulation and MMP9 dependency (Hamada et al. 2006; Iwakura et al. 2006; Suriano et al. 2008; Mackie et al. 2013). However, despite preclinical and observational evidence of a protective role for E2, randomized clinical trials have failed to translate the beneficial effects of E2 into a viable therapy for prevention of ischemic tissue injury, suggesting that estrogen-independent mechanisms might also contribute to sex disparity in cardio protection. Estrogen-independent aspects of sex dimorphism have, however, not gained similar attention and have been insufficiently studied. Evidence, however, exists that estrogen-independent mechanisms may also influence disease disparity observed between sexs. Innate differences in stroke risk exist between the sexes that are independent of hormone exposure (Rosamond et al. 2007, 2008). Animal studies on neuroprotection have shown clear sex differences in outcome using models where hormone levels are similar between the sexes (Nijboer et al. 2007). Thus, protection in females with greater homogeny of hormones between males and females further suggests an estrogen-independent regulation at work. Not much, however, is known about estrogen-independent sex differences in the context of stem cells. Female muscle-derived stem cells regenerate skeletal muscle more efficiently than male cells, but transplantation of male stem cells into female recipients or pre-treatment of male cells with E2 fails to yield comparable regeneration, implying that sex-related differences in stem cell function may not be entirely dependent on sex hormones (Deasy et al. 2007). In fact, sex has been considered as a predictor of methylation and differential methylation patterns between female and male-derived cells/tissues have been reported (Wiencke et al. 1999; Sarter et al. 2005; El-Maarri et al. 2007; Liu et al. 2010; Hartman et al. 2018). However, whether sex alters the DNA methylation patterns in age-matched male and female EPC/stem cells is not known.

While the amount of research linking epigenetics and CVD is climbing, studies on epigenetic regulation of sex-related differences in stem cells and their role in stem cell-based cardiac repair are scant. So far, a direct comparison of sex-specific epigenetic disparities, particularly sex hormone-independent mechanisms, has not been studied in the context of cardiac homeostasis and disease. We are currently investigating the estrogen-independent epigenetic mechanisms that regulate the differential cardiac reparative properties of bone marrow stem cells (BMSCs) post-MI. Briefly, we found that histone methyltransferase Ehmt2/G9a differentially regulates methylation of H3K9me3, affecting the angiogenic and inflammatory properties of BMSCs from male and female mice. However, we found significant differences in the genome-wide distribution of H3K9me3, as well as the associated inflammatory and reparative properties of BMSCs from female and ovariectomized (OVX) mice (manuscript under review).

## 6. Future directions and conclusion

It is evident from the research reviewed above that there are substantial differences in the levels of sex hormones, epigenetic regulators, and variability in the expression of several genes that regulate cardiac homeostasis and pathophysiology. Yet, the treatment regimens or approaches are similar for both men and women. The influence of genetic and environmental factors, such as lifestyle and diet, contribute to the epigenetic modifications that regulate the development and progression of CVDs. It is essential to gain a deeper insight into the differential roles of DNA methylation, histone modifications, and non-coding RNA in both sexes that lead to the incidence and progression of CVDs, dependent or independent of sex hormone regulation. These epigenetic modifications can serve as reliable biomarkers for CVD risk assessment. Profiling these epigenetic signatures in circulating blood cells or tissues may enable the development of noninvasive diagnostic tools, providing clinicians with early indicators of cardiovascular risk. Epigenome-wide Association Studies (EWAS) have the potential to identify specific epigenetic variations associated with CVDs. By comparing the epigenetic profiles of individuals with and without cardiovascular conditions, researchers can pinpoint novel epigenetic markers that may contribute to more accurate disease diagnosis and prognosis (Soler-Botija et al. 2019). Targeting specific epigenetic modifications implicated in CVD pathophysiology provides a promising avenue for therapeutic intervention. Small molecules, such as HDAC inhibitors or DNMT inhibitors, can modify aberrant epigenetic patterns, potentially reversing or slowing down the progression of cardiovascular conditions. A personalized medicine approach, tailoring treatments and interventions by understanding these sex-specific epigenetic disparities, can lead to effective strategies to manage or treat CVDs.

Many studies have investigated the role of sex hormones in cardiovascular pathologies; however, future cardiovascular research should also focus on sex-hormone-independent epigenetic mechanisms that can improve diagnostic and therapeutic strategies in men and women.

## Figures and Tables

**Table 1.** Epigenetic regulators of sex dimorphism in CVD.

Epigenetic regulators	Role in CVD	Sex dimorphism in CVD	References
DNMT1	Depletion of DNMT1 in rat model of heart failure improved cardiac function	Downregulation of ER*α* is associated with upregulation of DNMT1	Pinzone et al. 2004; Wu et al. 2020
DNMT3a	Knockout of DNMT3a in human cardiomyocytes resulted in low MYH7/MYH6 with dysregulation of HIF-1*α* Loss of DNMT3a in myeloid cells enhances inflammation	Undetermined	Madsen et al. 2020; Rauch et al. 2023
DNMT3b	Hypomethylation by DNMT3b dysregulation contributes to ischemic cardiomyopathy; 99% males and 1% female.	Undetermined	Tarazón et al. 2022
p300/CBP	Inhibition of p300/CBP reversed cardiac fibrosis, hypertrophy, and cardiac function in SIRT3 KO mice	Undetermined	Su et al. 2020
HDAC1	HDAC1-mediated deacetylation of H3K9Ac causes angiogenic dysfunction in cardiac endothelail cells	No differences observed in HDAC activity in regulating calcium handling and regulating cardiac contractility	Montgomery et al. 2007; Huang et al. 2022
HDAC2	Downregulated HDAC2 causes downregulation of GATA4, MEF2D, and SREBF2	No dimporphism observed	Montgomery et al. 2007
HDAC3	HDAC3 deletion results in cardiac hypertrophy and lipid accumulation in cardiomyocytes	HDAC3 knockdown increases triglycerides in female drosophila hearts	Montgomery et al. 2008; Ren et al. 2023
HDAC4	HDAC4 is upregulated in ischemic cardiac injury. Overexpression of HDAC causes cardiac hypertrophy	Undetermined	Zhang et al. 2018
HDAC5	HDAC5 is a repressor of angiogenesis and promotes cardiac hypertrophy	Male mice lacking HDAC5 were hypersensitive to pathological effects of MI, whereas female mice lacking HDAC5 were protected from adverse remodelling in MI	Urbich et al. 2009; van Rooij et al. 2010
Class II—HDACs4-9	Class II HDACs, particularly HDAC5 and 9, are signal-responsive repressors of cardiac hypertrophy	Female mice lacking HDAC5 or 9 upregulate ER*α*, resulting in protection from pathological remodelling post-MI	Zhang et al. 2002; van Rooij et al. 2010
SMYD1	SMYD1 disrupts cardiac differentiation and maturation, leading to embryonic lethality	Undetermined	Du et al. 2014
Dotl1	Dotl1significantly lower in human idiopathic dilated cardiomyopathy	Undetermined	Nguyen et al. 2011
miR-1, miR208	Overexpression of miR-1 in developing hearts decrease ventricular cardiomyocytes pool miR208 inhibition improves cardiac function in heart failure	No differences in miR-1 and miR208 expression between males and females with heart failure	Zhao et al. 2005
MHRT (lncRNA)	Downregulation of Mhrt causes cardiac hypertrophy	Undetermined	Montgomery et al. 2011
SNORD166 (snoRNA)	Enriched in the hypertrophic iPSC cardiomyocyte	Undetermined	James et al. 2021
CHAPIR (piRNA) HAAPIR (piRNA)	Regulated in cardiac hypertrophy by targeting METTL3 (m6A) Enhances acetylation on TFEC to induce cardiomyocyte death	Undetermined	Gao et al. 2020; Wang et al. 2022
Lionheart (lincRNA)	Upregulated in cardiac pathological remodeling	Undetermined	Kuwabara et al. 2020

**Note:** CVD, cardiovascular disease; HDAC, histone deacetylases; DNMT, DNA methyltransferase; iPSC, induced pluripotent stem cell.

## Data Availability

This manuscript does not report data.
